# Socio-Emotional, Behavior and Cognitive Disorders Associated with Exposure to Trace Metals: A Cross-Sectional Pilot Study in School-Age Children in Haut-Katanga Province

**DOI:** 10.3390/children12111505

**Published:** 2025-11-06

**Authors:** Eunice Bilonda Mbuyamba, Jean-Paul Ngoy Mande, Paul Musa Obadia, Celestin Banza Lubaba Nkulu, Vincent Haufroid, Benoit Nemery, Claude Mwamba Mulumba, Abdon Mukalay Wa Mukalay, Laurence Ris, Laurent Lefebvre, Daniel Okitundu Luwa E-Andjafono

**Affiliations:** 1 Department of Neurology and Psychiatry, Faculty of Medicine, University of Lubumbashi, Lubumbashi 1825, Democratic Republic of the Congo; ngoy.mande@unilu.ac.cd; 2 Department of Neuroscience, Faculty of Medicine and Pharmacy, University of Mons, 7000 Mons, Belgium; laurence.ris@umons.ac.be; 3 Centre de Recherche en Toxicologie et Santé Globale, Institut Supérieur des Techniques Médicales de Lubumbashi, Lubumbashi 4748, Democratic Republic of the Congo; musa.p.obadia@gmail.com; 4 Toxicology and Environment Unit, Department of Public Health, Faculty of Medicine, University of Lubumbashi, Lubumbashi 1825, Democratic Republic of the Congo; clubabankulu2017@gmail.com; 5 Louvain Centre for Toxicology and Applied Pharmacology, UC Louvain, 1200 Brussels, Belgium; vincent.haufroid@saintluc.uclouvain.be; 6 Department of Public Health and Primary Care, KU Leuven, 3000 Leuven, Belgium; 7 Department of Internal Medicine, Faculty of Medicine, University of Lubumbashi, Lubumbashi 1825, Democratic Republic of the Congo; mulumba.mwamba@unilu.ac.cd; 8 Clinical Epidemiology Unit, Department of Public Health, University of Lubumbashi, Lubumbashi 1825, Democratic Republic of the Congo; mukalay.mukalay@unilu.ac.cd; 9 Department of Cognitive Psychology and Neuropsychology, Faculty of Psychology and Educational Sciences, University of Mons, 7000 Mons, Belgium; laurent.lefebvre@umons.ac.be; 10 Neuro-Psychopathological Centre, Faculty of Medicine, University of Kinshasa, Kinshasa B.P. 825, Democratic Republic of the Congo; daniel.okitundu@unikin.ac.cd

**Keywords:** schoolchildren, NEPSY-II, SDQ-Tutor, trace metals, DRC

## Abstract

**Introduction**: Trace metals can negatively impact biological functions and brain development. Cognitive and neurobehavioral disorders in children are poorly documented in Haut-Katanga Province, an area with significant and multiple exposures to trace metals that can lead to the exacerbation of cognitive and behavioral disorders. **Objective**: This study aimed to characterize the behavior of schoolchildren linked to their cognitive performance in urban and rural environments. **Methods**: A cross-sectional pilot study was conducted on 52 schoolchildren aged 6 to 11 (22 from rural areas presumed less exposed to metals and 30 from Lubumbashi, DRC). This study employed NEPSY-II tests, the Strengths and Difficulties Questionnaire (SDQ-Tutor), ENA 2020 software and trace metal spectrometry assays. Statistical tests were carried out with SPSS-20 and Stata-18. **Results**: Our findings revealed a correlation between children’s malnutrition and low mother’s education. The “total difficulties score” was more prevalent in rural areas (73%) compared to urban settings (37%) *p* < 0.05), in contrast to the “negative impact of difficulties” (59% versus 57%, *p* > 0.05). Urban children demonstrated superior cognitive performance, particularly in “facial affect recognition” (8 versus 4, *p* = 0.013) and “inhibitory control” (6.5 versus 3, *p* = 0.032). As-U(urine), Cd-B(blood), Hg-B, Mo-U, Ni-U, Pb-U, Pb-B and Sb-U were elevated compared to references. In general, urban areas had higher metal levels than rural areas. Blood and urine metals showed a complex and significant relationship with behavioral difficulties or cognitive performance. **Conclusions**: The observed behavioral issues, cognitive performance deficits and their association with nutritional deficiencies and trace metal exposure suggest a multifactorial neurodevelopmental origin. These findings highlight the need for further research in the region.

## 1. Introduction

The Haut-Katanga province is part of the Copperbelt region, located on the border between northern Zambia and the southern Democratic Republic of Congo (DRC). This region is globally known for its copper and cobalt mining industries. However, the poor regulation of mining activities has been identified as a major source of environmental pollution and human exposure to trace metals (TMs) in the area [1]. Acute and chronic exposures to trace metals can negatively impact biological functions and brain development, leading to adverse effects on the nervous, skeletal, endocrine, immune and circulatory systems [2].

Epidemiological studies conducted in Lubumbashi [3,4] have reported nutritional and cognitive impairments among children exposed to trace metals. Similarly, research in the former Katanga province has identified contaminated vegetables, fish and dust ingestion as the primary sources of trace metals in children [5]. Several factors contribute to children’s vulnerability, including their tendency to play on the ground and put objects in their mouths, their higher food intake per unit of body weight and their faster absorption rates compared to adults. Additionally, their nervous and immune systems are still maturing [6,7]. 

Approximately 5% to 15% of children worldwide are affected by developmental disorders [8]. A study conducted in six sub-Saharan African countries, including Uganda, Nigeria, South Africa, Ethiopia, the DRC and Kenya, reported a prevalence of behavioral disorders in children ranging from 12% to 33% [9]. In the nervous system, trace metal accumulation disrupts metabolism, causing behavioral, socio-emotional and cognitive disorders [2]. For example, exposure to lead has been shown to impair cognitive function and contribute to various neurodevelopmental disorders [10]. In the DRC, studies have described children’s behavioral disorders associated with lead [11], zinc and copper [12] and cognitive impairment related to exposure to multiple TMs in the Haut-Katanga province [4].

Environmental exposure to metals and neurotoxicants, as well as malnutrition, is likely to affect children’s behavior before cognitive disorders manifest. Given the reported cognitive impairments in school-aged children in Haut-Katanga, an area heavily impacted by metal pollution, it is important to investigate whether behavioral disorders are associated with these cognitive deficits.

This study aimed to assess the socio-emotional, behavior and cognitive performances of school-aged children in metal-polluted areas of Haut-Katanga.

To our knowledge, this is the first study in the region to combine behavioral, socio-emotional and cognitive assessments with the biomonitoring of trace metals, thereby providing a unique perspective on children’s health outcomes. This study further strengthens its originality by comparing two distinct groups of school-aged children—those from metal-polluted areas and those from less exposed areas—allowing for a more robust evaluation of the impact of environmental exposure on child development.

## 2. Methods

### 2.1. Study Design and Setting

This cross-sectional pilot study, conducted between April and July 2019, included school-aged children (6 to 11 years old) who were selected through convenience sampling from two areas: Lubumbashi, an urban area known for high levels of pollution (urban area, UA), and Kasongo village, a rural control area located approximately 50 km from Lubumbashi (rural area, RA).

Children were eligible if they had attended the same school for at least two years before the survey, to ensure their long stay in that environment. This was convenience sampling. The rural comparison group was recruited using the same criteria as the urban group, based on maternal and paternal age at childbirth, as well as maternal education level, to be considered as potential confounding factors in interpretation of results. Written consent was obtained from parents, and oral assent was obtained from children.

**Figure 1:**
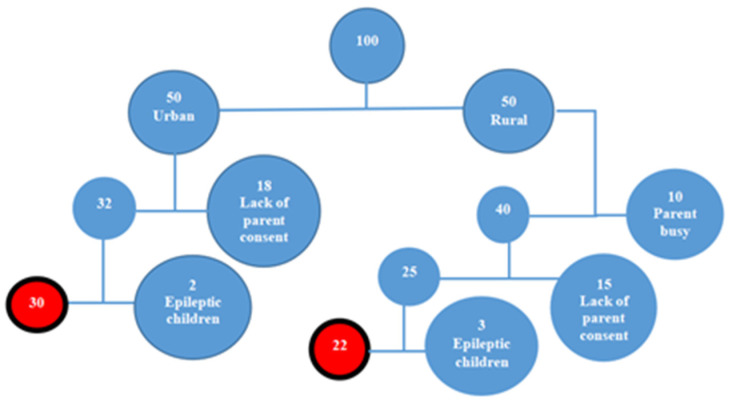
Schematic conception of child recruitment.

Due to logistical constraints and the high cost of analysis, consent was obtained for 32 children from the urban area and 25 from the rural area. However, three children from the rural area and two from the urban area were excluded due to a history of epilepsy, resulting in a final sample of 52 participants (30 from the urban area and 22 from the rural area) (Figure 1).

### 2.2. Data Collection

#### 2.2.1. Socio-Demographic Characteristics

A standardized questionnaire was used to collect socio-demographic information for all children and their parents. Each child’s nutritional status was also assessed using ENA 2020 for SMART [13], which focuses on age, weight and height.

#### 2.2.2. Behavioral Assessment

We administered the tutor version of the Strengths and Difficulties Questionnaire (SDQ-Tutor) to assess emotional symptoms, conduct problems, hyperactivity, peer relationship issues and prosocial behavior [14]. The SDQ contains 25 items divided into five scales, each scored on a 0–10 scale. Based on these scores, behavior was classified as “normal,” “borderline” or “abnormal.” This study dichotomized the results into “normal” and “behavioral problems” to facilitate analysis. The latter combined both borderline and abnormal categories.

#### 2.2.3. Neurocognitive Assessment

We used the French version of the NEPSY-II [15] to assess children’s cognitive performance across six domains: (1) attention and executive function, (2) memory and learning, (3) language, (4) visuospatial processing, (5) sensorimotor function and (6) social perception. Multiple scores were calculated for each test, and calibrated scores were obtained for each child, as described in the NEPSY-II documentation [16].

We acknowledged that NEPSY-II norms are not adapted to the Congolese context, which may influence cross-group comparisons.

#### 2.2.4. Sample Collection and Analysis

Urine samples were collected in 40 mL polystyrene bottles. Blood samples (4 mL) were taken from the brachial vein using BD Vacutainer^®^ K2EDTA tubes (Becton Dickinson, Franklin Lakes, NJ, USA). The toxicology laboratory team from the University of Lubumbashi collected samples, kept them frozen at −20 °C and then transferred them to Leuven, Belgium, where they were stored at −80 °C until analysis.

All samples were analyzed at the Leuven Centre for Applied Toxicology and Pharmacology using inductively coupled plasma mass spectrometry (ICP-MS) for metal detection. Eight elements were measured in blood [17], and twenty-five elements were measured in urine [18].

International pediatric reference values from Belgium and Canada were used due to lack of local norms [19,20].

### 2.3. Statistical Analysis

Initial analyses included comparisons of socio-demographic, behavioral and cognitive characteristics between the two study groups using Student’s *t*-tests, Mann–Whitney U tests, median and geometric mean. Spearman’s correlation coefficients were calculated to assess the relationships between age, nutritional status and behavioral and cognitive scores. Statistical significance was set at *p* ≤ 0.05.

Effect sizes and 95% confidence intervals for the main comparisons were used to improve interpretation, but this study did not adjust for multiple testing due to its pilot nature, and as such all associations should be considered exploratory.

### 2.4. Ethical Considerations

The study protocol was approved by the Medical Ethics Committee of the University of Lubumbashi (UNILU), under approval number UNILU/CEM/036/2019.

## 3. Results

### 3.1. General Characteristics (Table 1)

Among the children in the urban area (UA), girls accounted for 57%, while in the rural area (RA) the sex ratio was 1:1. The children’s ages ranged from 6 to 11 years, with a mean (±SD) of 9.0 ± 1.6 years for urban children and 8.6 ± 1.5 years for rural children. The reported birth weight of children in the rural area was significantly higher than that of urban children (*p* = 0.001). But rural children had a statistically significant worse nutritional status (HAZ and WAZ) compared to those living in urban areas [13]. In addition, children in the urban area had mothers with higher levels of education, compared with mothers of children in the rural area (Table 1).

### 3.2. Biological Monitoring of Metals (Table 2)

The concentrations of several trace metals (arsenic As-U, cadmium Cd-B and mercury Hg-B for Canadian inquiry, rural molybdenum Mo-U, nickel Ni-U, lead Pb-U, lead Pb-B, Sb-U), were elevated when compared with reference values obtained in industrially developed countries [19,20]. The Cd-B study values were higher only for urban children, compared with the references of Bohn et al. In contrast, the latter reference of Hg-B was higher than the study’s urban and rural values. For several trace metals, concentrations were, as expected, higher in blood or urine among urban children than rural children. In blood, exposed children had higher geometric mean concentrations than controls for cobalt (*p* < 0.001), cadmium (*p* < 0.0001) and mercury (*p* < 0.0001). In urine, geometric mean concentrations with creatinine corrections were higher for lithium (*p* < 0.001) among the urban area (UA) compared to the rural area (RA). In the urine of rural children, the molybdenum (*p* < 0.008) and nickel (*p* < 0.025) values were higher than the urban ones.

### 3.3. Behavioral Characteristics and Cognitive Assessment

Table 3 shows no statistically significant differences between the groups in the conduct problems score, relationship score, impact score, or prosocial score. However, children in the rural area (RA) had higher scores, closer to abnormal values, and significant differences compared to those in the urban area (UA) for hyperactivity (3 vs. 5; *p* = 0.018) and total difficulties (11.5 vs. 16.5; *p* = 0.018). A difference was also observed in the emotional score (2 vs. 4; *p* = 0.021).

UA values were statistically higher than the RA values for inhibition (IND: 6.5 vs. 3; *p* = 0.032), facial affect recognition (FRA: 8 vs. 4; *p* = 0.013), delayed word list memory (DLWLM: 6 vs. 3; *p* < 0.05), categorization (“CA Total”: 14 vs. 10.5; *p* = 0.008) and list interference (“IM total”: 12 vs. 4; *p* = 0.002).

Table 4, with SDQ-Tutor, shows a statistically significant (*p* = 0.01) difference for the rate of behavioral problems only in total difficulties (UA 37% vs. RA 73%; *p* = 0.010). Without a statistically significant difference compared with the UA (Table 3 SDQ-Tutor), the RA had higher rates of other behavioral problems: relationship problems (68% vs. 57%), Impact (59% vs. 57%) and emotional disorders (55% vs. 30%). Overall, the lower rate of behavioral problems was 37% in the UA, and the higher rate was 73% in the RA.

### 3.4. Relationship Between Behavioral Problems, Sex, Age and Nutritional Status

There were no differences between the scores of girls and boys, so the values for both sexes were combined.

In the urban area (UA), statistically significant negative correlations were found between age and total behavioral difficulties scores (r = −0.549; *p* = 0.002), emotional scores (r = −0.494; *p* = 0.006), hyperactivity scores (r = −0.538; *p* = 0.002), relational scores (r = −0.410; *p* = 0.024) and the prosocial behavior score (r = −0.443; *p* = 0.014). In contrast, no statistical correlations were observed for these variables in the rural area (RA) group.

Overall, total behavioral difficulties scores correlated negatively with the HAZ nutritional status (r = −0.284; *p* = 0.042). Prosocial behavior scores correlated positively with the HAZ (r = 0.28; *p* = 0.045) and WAZ (r = 0.283; *p* = 0.042).

### 3.5. Relationship Between Behavioral Problems and Cognitive Performances

In Table 5 we present the statistically significant results. In the RA group, we found a negative correlation between the impact of difficulties (impact scores) and the inhibition scores (IND). Also, impact scores were positively correlated to TPRC (digital dexterity performance) scores.

In the UA group, negative correlations were also found between facial recognition scores (FRA) and total difficulties scores and hyperactivity scores. On the other hand, positive correlations were observed between the comprehension of instruction (CC) and conduct problems, emotional scores and their impact score. The emotional score was negatively correlated with the inhibition score (IND) and word list interference (IM). Also, the total difficulties score was negatively correlated with the inhibition score (IND).

Finally, high scores in conduct problems and hyperactivity were negatively associated with digital dexterity performance (TPRC).

### 3.6. Relationship Between Behavioral Problems and Trace Metals Exposition

Overall, Table 6 shows that total behavioral difficulties scores correlated positively with corrected urinary levels of aluminum (Al-U) and molybdenum (Mo-U) and negatively with blood levels of cadmium (Cd-B) and mercury (Hg-B).

Emotional scores negatively correlated with blood levels of Hg-B and Cd-B, as well as with corrected urinary levels of titanium (Ti-U).

Hyperactivity scores positively correlated with corrected urinary levels of Mo-U, Al-U and Ti-U.

Conduct problems scores correlated positively with corrected urinary aluminum levels (Al-U), manganese (Mn-U and Ti-U).

Relationship scores were negatively correlated with Hg-B, selenium (Se-U) and chromium (Cr-U).

Prosocial scores correlated positively with blood levels of cobalt (Co-B) and cadmium (Cd-B) and corrected urinary levels of titanium (Ti-U) then negatively with copper (Cu-U).

Finally, the total impact score correlated positively with blood lead levels (Pb-B) and then negatively with Hg-B and Co-U.

Some of these correlations remain significant when considering only the UA group, as most of these metals were detected at higher levels in the UA compared to the RA.

## 4. Discussion

This study aimed to characterize children’s behavior in relation to cognitive performance and exposure to trace metals in two neighborhoods: the urban area with high metal exposure and the rural area presumed to have a lower exposure. We focused on behavioral characteristics, cognitive performance, the relationship between behavioral characteristics and cognitive performance and then the association between behavioral characteristics and exposure to trace metals.

Compared to urban children, rural children had higher birth weights but a lower HAZ, WAZ and mother’s education. The DRC DHS (2023–2024) found a low HAZ and low WAZ in rural settings [21].

### 4.1. Behavioral Characteristics

This high frequency of behavioral problems, observed in this study using SDQ-Tutor (37 to 73% overall), is in line with what was found in Latvia in a metropolitan area among children aged 2 to 17, with frequencies of 60.2% for relationship problems, 49.3% for emotional disorders and 47.9% for social conduct disorders [22].

In our study, the significantly higher frequency of problems relating to “total difficulties” of rural children (73%) compared with urban children (37%) could be explained by the poorer nutritional status in rural areas [21]. In our study, total behavioral difficulties scores correlated negatively with the HAZ nutritional status. Malnutrition is a known risk factor for behavioral problems in children [23].

### 4.2. Cognitive Performances

Table 2 showed that children in the urban environment performed cognitively (*p* < 0.05) better than those in the rural environment in terms of attention and executive functions measured by categorization and the degree of inhibition, social perception assessed by facial recognition of affect and learning memory in relation to word list interference and delayed word list memory. The difference in cognitive scores between the two environments could be explained by the negative consequences of malnutrition on cognition [24]. In this study, the children in the rural environment showed deteriorated nutritional status in terms of statural and weight development and growth. The parents’ level of education is a determinant of the cognitive scores assessed with NEPSY-II [24]. Several studies support the theory that a high level of parental education has an impact on children’s cognitive level, with a particular emphasis on the mother’s level of education [25].

### 4.3. Cognitive Performance and Behavioral Characteristics

The present study revealed several correlations between cognitive performance and the behavioral characteristics of children. Facial affect recognition scores were negatively correlated with total difficulty scores. This aligns with the existing literature, which indicates that emotional and behavioral disorders, as assessed by the Strengths and Difficulties Questionnaire—Teacher Version (SDQ-T), are associated with poorer performance in facial affect recognition [26].

Lower inhibition performance was associated with higher emotional behavior scores among urban children and with a greater negative impact of behavioral difficulties among rural children. These findings underscore the interconnectedness of attention, executive functions and emotional and behavioral regulation [27].

Relational difficulties and hyperactivity scores were negatively correlated with social cognition and facial affect recognition, highlighting the critical role of social cognition and the theory of mind in interpersonal relationships and social behavior [28].

Furthermore, higher scores for social conduct problems, hyperactivity and the negative impact of behavioral difficulties were significantly associated with poorer performance on digital dexterity tasks (TPRC), suggesting potential prefrontal dysfunction in children exhibiting hyperactivity and conduct disorders [29].

### 4.4. Behavioral Characteristics and Exposure to Trace Metals

The presence of metals that have no biological role in the human body in the urine and blood of children proves the exposure to trace metals in Haut-Katanga province, which requires further in-depth study (Pb, As, Cd, Hg, Ti) [1,6].

As-U; urban Cd-B (references of Bohn et al.); Hg-B (for Canadian inquiry); and rural Mo-U, Ni-U, Pb-U, Pb-B and Sb-U were elevated when compared to the reference values reported by Bohn et al. [19,20].

Moreover, it was found that blood levels of cobalt, cadmium and mercury and corrected urine levels of lithium were higher in the UA than in the RA. Contrarily, Mo-U and Ni-U in the RA were higher than in the UA, as reported elsewhere [30].

Table 6 reveals that prosocial behavior in the UA was associated positively with Co-B, Cd-B and Ti-U but negatively with Cu-U. Urban relational scores and the negative impact of behavioral difficulties decreased with higher Hg-B levels, while Pb-B levels increased with the negative impact of behavioral difficulties. Our data showed that the exposure of children to trace metals in both rural and urban environments was associated with behavioral problems in a complex way (total difficulties, emotional, hyperactivity, relationship, conduct problems, prosocial and impact scores), as reported elsewhere [31].

Al-U levels were associated with higher scores of hyperactivity and conduct problems. Indeed, aluminum accumulation in the body, even at low doses, has long-term neurotoxic effects for neurobehavioral expression [32]. It is important to note that all trace metals in excess in the children’s blood (Cd, Pb, Co) impact behavioral scores. The negative correlation between Hg-B and behavioral scores may reflect complex inter-metal interactions rather than a protective effect. The urinary elimination of cobalt and chromium reduced the negative impact scores for behavioral difficulties, which probably reflects a behavioral neurotoxicity of these metals. It is known that urinary concentrations of cobalt and chromium are reliable indicators of exposure to these metals [33]. The presence of titanium in children’s urine with emotional and behavioral disorders suggests exposure to this metal, which has no biological role in the body.

In the context of multiple exposures, which implies interactions between different metals, this relationship with behavioral problems should be investigated in future studies. Each development activity (e.g., mining) or health intervention (promotional, preventive, curative, etc.) must consider our results.

This study highlights the need for integrated actions addressing trace metal exposure, malnutrition and education in Haut-Katanga children. Future research should expand to larger, longitudinal studies to confirm these associations. Meanwhile, school-based nutritional programs, environmental monitoring and awareness campaigns are essential to reduce exposure and protect children’s neurodevelopment in the region.

## 5. Strengths and Limitations

The strengths of this study include its originality as the first epidemiological study to explore behavioral characteristics of children using validated behavioral and neurocognitive assessment tools and the characterization of trace metal exposure by blood and urinary biomonitoring. In this context, interventions related to trace metals, behavioral and cognitive aspects of school-age children will have to take into account our results. Nevertheless, we also acknowledge several limitations: firstly, the cross-sectional nature of the study; secondly the small sample size; thirdly, we were not able to determine cerebral morbidity, prenatal maternal stress and maternal depression; and fourthly, there was no predictor of negative impact. The results obtained are not entirely generalizable in another context without precautions.

## 6. Conclusions

The association of behavioral disorders with age, nutritional disorders, trace metals and cognitive performances in children in Haut-Katanga indicates the neurodevelopmental and multifactorial origin of these disorders, probably linked to multiple exposures to trace metals and the combined effect of malnutrition and metal exposure. Further studies are required to explore these complex relationships.

## Figures and Tables

**Table 1:** Socio-demographic characteristics.

Parameters	Urban Children n = 30	Rural Children n = 22	*p*
Age (years)	9.0 ± 1.6	8.6 ± 1.5	0.37
Female (n, %)	17 (57%)	11 (50%)	0.68
Male (n, %)	13 (43%)	11 (50%)	
Weight (kg)	28.8 ± 4.9	23.5 ± 4.9	0.001
Height (cm)	132.2 ± 8.99	121.27 ± 10.95	0.001
HAZ	−0.23 ± 1.25	−1.45 ± 1.68	0.002
WAZ	0.03 ± 1.13	−1.00 ± 1.31	0.002
Head circumference (cm)	51.9 ± 1.8	51.4 ± 1.8	0.37
Brachial perimeter (cm)	18.0 ± 1.2	16.9 ± 1.3	0.05
Birth weight (g)	3119 ± 438	3333 ± 624	0.001
Primary school starting age	5.85 ± 0.755	5.95 ± 0.745	0.633
Mother’s age at childbirth (years)	30.22 ± 6.15	25.73 ± 6.31	0.015
Father’s age at child’s birth (years)	37.56 ± 7.92	34.09 ± 6.49	0.106
Mother’s educational level			
Illiterate	0 (0%)	5 (23%)	<0.05
Primary	0 (0%)	9 (41%)	
Secondary	24 (89%)	8 (36%)	
University	3 (11%)	0 (0%)	

Legend: Values reported are mean ± SD for quantitative variables or n (%) for qualitative variables; *p*-values based on Fisher Exact or Student *t*-test; HAZ = Height for Age Z-Score; WAZ = Weight for Age Z-Score; and SD = Standard Deviation.

**Table 2:** Biological monitoring of trace metal elements.

TM	Urban Children n = 30	Rural Children n = 22	Urban vs. Rural	Health Canadian (August 2010) [20]	Bohn et al., 2023 [19]
	Geometric Mean (IC 95%)		*p*	Geometric Mean	Pediatric Limit Reference
Al-U (µg/g)	18.3 (13.4–24.9)	19.8 (14.1–27.8)	0.64		
As-U (µg/g)	33.9 (24.2–47.8)	34.9 (27.1–44.9)	0.51	14.8	
Ba-U (µg/g)	3.04 (2.29–4.04)	1.71 (0.98–2.97)	0.53		
Cd-U (µg/g)	0.31 (0.26–0.36)	0.31 (0.23–0.39)	0.78	0.34	
Cd-B (µg/dL)	0.05 (0.04–0.06)	0.02 (0.01–0.02)	<0.001	0.01	0.03
Co-U (µg/g)	6.68 (4.83–9.25)	5.34 (3.92–7.27)	0.19		
Co-B (µg/dL)	0.09 (0.06–0.13)	0.04 (0.03–0.05)	<0.001		
Cr-U (µg/g)	0.31 (0.25–0.37)	0.23 (0.19–0.26)	0.07		
Cu-U (µg/g)	16.2 (13.9–18.9)	14.4 (11.3–18.5)	0.66	16.1	
Hg-B (µg/dL)	0.19 (0.15–0.24)	0.13 (0.12–0.14)	<0.001	0.03	0.19
Li-U (µg/g)	22.9 (18.6–28.4)	12.3 (10.8–14.2)	0.001		
Mn-U (µg/g)	1.15 (0.72–1.84)	1.02 (0.55–1.88)	0.67	0.12	
Mn-B (µg/dL)	0.92 (0.67–1.25)	1.17 (1.03–1.32)	0.14	9.86	
Mo-U (µg/g)	77.6 (67.2–89.6)	106 (76.4–147)	0.01	87.0	
Ni-U (µg/g)	3.08 (2.51–3.77)	4.71 (3.51–6.33)	0.03	2.00	
Pb-U (µg/g)	3.75 (2.87–4.89)	3.24 (2.56–4.10)	0.21	0.56	
Pb-B (µg/dL)	6.31 (4.05–9.82)	8.41 (6.74–10.5)	0.40	0.90	1.14
Sb-U (µg/g)	0.10 (0.08–0.12)	0.09 (0.08–0.12)	0.80	0.08	
Se-U (µg/g)	19.2 (16.3–22.5)	19.3 (16.8–22.1)	0.79	92.8	
Sn-U (µg/g)	0.14 (0.09–0.19)	0.12 (0.08–0.18)	0.50		
Te-U (µg/g)	0.16 (0.12–0.19)	0.14 (0.11–0.17)	0.38		
Ti-U (µg/g)	2.73 (2.25–3.29)	3.19 (2.57–3.95)	0.25		
Tl-U (µg/g)	0.19 (0.15–0.26)	0.18 (0.14–0.21)	0.21		
V-U (µg/g)	0.24 (0.13–0.35)	0.24 (0.17–0.32)	0.54		
Zn-U (µg/g)	532 (454–625)	477 (381–595)	0.50	451	

Legend: U = urine by creatinine, B = blood and *p* = *p*-value by Mann–Whitney U test.

**Table 3:** Behavioral and cognitive characteristics.

	Urban Children n = 30			Rural Children n = 22			
NEPSY-II	P25	Median	P75	P25	Median	P75	*p*
CA Total	10	14	16	7.25	10.5	11.8	0.008
IND Comp	3.75	6.5	9.25	1	3	7	0.032
FRA Total	4	8	9	2	4	7	0.013
DLWLM	4	6	8	2	3	3.75	<0.01
IM Total	4	12	14	2	4	5.5	0.002
Total Difficulties	6.75	11.5	15.5	12.75	16.5	19	0.009
Emotional Sc.	1	2	4	1.75	4	5	0.021
Conduct Problems Sc.	1.75	2.5	5	1	2.5	4	0.465
Hyperactivity Sc.	2	3	5	3.75	5	7	0.018
Relationship Sc.	2	3	5	2	3.5	5	0.566
Prosocial Sc.	6	8.5	10	5.75	7.5	9	0.218
Impact Sc.	0	1	4	0	2	3	0.977

Legend: CA total = categorization; IND = inhibition; FRA = facial affect recognition; DLWLM = delayed list memory; IM = word list interference; Sc = score; *p* values by Mann–Whitney U tests.

**Table 4:** Behavioral problem rates with SDQ-Tutor.

	Urban Children n= 30		Rural Children n= 22		
SDQ-Tutor	Normal	With Disorders	Normal	With Disorders	*p*
Total Difficulties, n (%)	19 (63)	11 (37)	6 (27)	16 (73)	0.010
Emotional Sc., n (%)	21 (70)	9 (30)	10 (45)	12 (55)	0.075
Conduct Problems Sc., n (%)	15 (50)	15 (50)	12 (55)	10 (45)	0.746
Hyperactivity Sc., n (%)	25 (83)	5 (17)	14 (64)	8 (36)	0.105
Relationship Sc., n (%)	13 (43)	17 (57)	7 (32)	15 (68)	0.399
Prosocial Sc., n (%)	25 (83)	5 (17)	18 (82)	4 (18)	0.887
Impact Sc., n (%)	13 (43)	17 (57)	9 (41)	13 (59)	0.861

Scores as determined by the SDQ questionnaire (%); *p*-values by Khi-square.

**Table 5:** Spearman correlation coefficients between cognitive performance and behavioral SDQ scores.

	Total Difficulties	Emotional Scores	Conduct Problems Scores	Hyperactivity Scores	Relational Scores	Prosocial Behavior	Impact Score
CA UA	−0.04	−0.15	−0.15	0.09	−0.12	−0.09	0.16
CA RA	−0.15	−0.31	−0.31	−0.23	0.43	−0.06	0.01
RAHO UA	−0.28	−0.30	−0.30	−0.08	−0.26	−0.24	−0.16
RAHO RA	−0.43	−0.43	−0.43	0.17	−0.19	0.22	−0.23
IND UA	−0.35 *	−0.35 *	−0.35	−0.45	−0.00	−0.17	−0.09
IND RA	−0.18	−0.18	−0.18	−0.07	0.25	0.20	−0.44 *
CC UA	0.49 *	0.45 *	0.45 *	0.39	0.34	−0.02	0.46 *
CC RA	0.24	0.11	0.11	0.38	−0.08	−0.26	0.12
FRA UA	−0.52 *	−0.33	−0.33	−0.49 *	−0.56	−0.12	−0.26
FRA RA	−0.05	0.06	0.06	0.09	−0.01	0.19	−0.25
PG UA	−0.22	−0.25	−0.25	−0.16	−0.29	0.12	−0.12
PG RA	−0.16	0.11	0.11	−0.03	−0.12	−0.11	−0.18
IP UA	0.06	0.13	0.13	−0.15	0.17	−0.20	0.02
IP RA	−0.06	−0.05	−0.05	0.05	−0.16	−0.07	−0.23
DLWLM UA	−0.39	0.21	0.21	0.22	−0.02	−0.17	0.15
DLWLM RA	−0.12	−0.15	−0.15	−0.19	−0.04	0.03	−0.04
IM UA	−0.35	−0.37 *	−0.37	−0.21	−0.27	−0.15	−0.10
IM RA	−0.10	−0.38	−0.36	−0.31	−0.22	0.33	−0.26
TPRC UA	−0.26	−0.42	−0.42 *	−0.47 *	0.01	0.16	−0.26
TPRC RA	−0.17	0.35	0.35	0.33	−0.29	−0.11	0.05 *

Legend: CA total = categorization; RAHO = associated response and clock; IND = inhibition; CC = comprehension of instructions; FRA = facial recognition of affect; PG = geometric puzzle; IP = image puzzle; DLWLM = delayed list memory; IM = word list interference; TPRC = tapping; UA = urban exposed environment children; RA = rural less exposed presumed children; and * = *p* < 0.05.

**Table 6:** Spearman correlation between blood or urine metal concentrations and SDQ scores.

SDQ Parameters	Metals	Urban Children	Rural Children	All
Total difficulties	Cd-B			−0.33 *
	Hg-B			−0.36 *
	Mo-U			0.37 *
	Al-U			0.29 *
Emotional scores	Hg-B			−0.35 *
	Ti-U	−0.37 *		−0.31 *
	Cd-B			−0.30 *
Hyperactivity scores	Mo-U	0.53 *		0.39 *
	Al-U			0.32 *
	Ti-U	0.44 *		0.31 *
Relationship scores	Hg-B			0.32 *
	Se-U	−0.44 *	−0.51 *	
	Cr-U		−0.49 *	
Conduct problems scores	Mn-U	0.42 *		
	Al-U	0.42 *		0.37 *
	Ti-U	0.50 *		
Prosocial Scores	Co-B	0.43 *		
	Cd-B			0.39 *
	Ti-U			0.37 *
	Cu-U	−0.45 *		0.37 *
	Tl-U	0.41 *		
Impact scores	Hg-B	−0.39 *		
	Pb-B	0.48 *		0.35 *
	Co-U		−0.48 *	

Legend: * = *p* < 0.05 level of significance; the values correspond to the correlation coefficient; B = blood in µg/dL; and U = urine value corrected by creatinine in µg/g.

## Data Availability

The data presented in this study are available on request from the corresponding author. The data are not publicly available due to privacy or ethical restrictions.
